# Regulation of nociception threshold by norepinephrine through adrenergic α2 receptor in rat models of Parkinson's disease

**DOI:** 10.1111/cns.14446

**Published:** 2023-09-18

**Authors:** Qing Gao, Yingying Zhang, Xiaoying Wang, Rui Wang, Limei Zhang

**Affiliations:** ^1^ Department of Neurology The Second Affiliated Hospital of Harbin Medical University Harbin China

**Keywords:** GFAP, nociception threshold, norepinephrine, Parkinson's disease, α2‐adrenergic receptors

## Abstract

**Background:**

The mechanism of pain symptoms in Parkinson's disease (PD) is unclear. Norepinephrine (NE) regulates neuropathic pain through ascending and descending pathways. However, the loss of NE neurons in the brain of patients with PD is obvious, it is speculated that NE is involved in the occurrence of PD pain symptoms.

**Aims:**

To investigate the effect of NE on the activation of brain cells through adrenergic α2 receptor, so as to regulate the nociception threshold in a 6‐OHDA‐induced animal model of PD.

**Methods:**

PD rat model was established by 6‐OHDA injection (6‐OHDA group). DSP‐4 (or anti‐DBH‐saporin) was used to reduce the NE level of the PD rat brain. The heat sensitivity threshold (HST) and pressure withdrawal threshold (PWT) were measured. Tyrosine hydroxylase and NE in rat brains were detected by Elisa. The percentage of GFAP‐positive cells in the prefrontal cortex, cingulate gyrus and striatum of rats was detected by immunohistochemistry and immunofluorescence. GFAP protein was semiquantified by method of western blot. Then yohimbine and guanfacine were used to increase the NE level in PD rats, and the above experimental changes were observed after drug application.

**Results:**

The contents of NE in the brain of 6‐OHDA‐induced PD rats were lower than that of control group. After DSP‐4 (or anti‐DBH‐saporin) injection, PD rats showed the lowest NE level (compared with 6‐OHDA group, *p* ≤ 0.05), and after yohimbine and guanfacine were applied to 6‐OHDA group, the contents of NE increased in the prefrontal cortex of rats. The HST and PWT of 6‐OHDA group were significantly lower than those of control group, and after DSP‐4 (or anti‐DBH‐saporin) injection, the HST and PWT of rats were lower than those of 6‐OHDA group, and after the administration of yohimbine and guanfacine, both HST and PWT were significantly increased. GFAP‐positive cells increased in prefrontal cortex and anterior cingulate gyrus of 6‐OHDA group rats, and more significantly increased after DSP‐4 (or anti‐DBH‐saporin) injection, and significantly reduced after yohimbine and guanfacine were used.

**Conclusions:**

The change of norepinephrine content can affect the activation of prefrontal and cingulate gyrus glial cells and participate in the regulation of nociception threshold in PD rats. Adrenergic α2 receptor agonist and central presynaptic membrane α2 receptor blocker both affect cell activation and improve hyperalgesia.

## Graphical abstract

This study revealed that norepinephrine neuronal damage can lower the nociception threshold of 6‐OHDA‐induced PD rats, and the rats showed an increased proportion of GFAP positive cells in the prefrontal lobe and cingulate gyrus. Adrenergic α2 receptor agonist and central presynaptic membrane α2 receptor blocker both decreased the ratio of GFAP positive cells and improve nociception threshold.

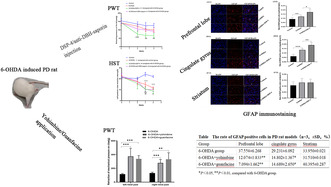

## 1 INTRODUCTION

Pain is a common nonmotor symptom in Parkinson's disease (PD) with an incidence of 50%–80%.^1^ Previous studies have classified the pain symptoms into PD‐related pain and non‐PD‐related pain. Among them, PD‐related pain symptoms have received more attention, which seriously affects the patients' quality of life. The central mechanism of pain symptoms in PD is complex. Pathological damage to the pain processing area may result in reduced pain symptoms and pain threshold.

The main biochemical change in patients with PD is the reduction of monoamine neurotransmitters, including dopamine, norepinephrine (NE) and serotonin. Previous studies have paid much attention to the relation of dopamine with PD pain symptoms.^2^ But levodopa does not work for all the pain types in PD patients. Duloxetine, however, may solve some patients' feeling of pain.^3^, ^4^ It indicated that NE and serotonin may be involved in the regulatory mechanism of neuropathic pain symptoms in PD.^5^

Noradrenergic neurons are reduced by 90% in the locus coeruleus of PD patients.^6^, ^7^ Clinical studies have also found that Magnetic Resonance Imaging (MRI) sensitive to neuromelanin can indicate reduced or even disappeared signal in the locus coeruleus area in PD patients.^8^ Norepinephrine cell population in locus coeruleus is located on the dorsal pons. The nerve fibers emitted by this group of cells spread almost throughout the central nervous system and are involved in the regulation of stress, emotional memory, motor, autonomic function and pain.^9^, ^10^ It has been demonstrated that activation and inactivation of projection fibers in the locus coeruleus affect the response to acute and chronic pain symptoms.^11^ These raised concerns about the role of norepinephrine (NE) in PD pain symptoms.

It has been reported that norepinephrine can regulate the electrical activity of the cortico‐striatum thalamic circuit,^4^ and the absence of NE may aggravate the paresthesia in PD patients. The most common finding after activation of the α2‐adrenergic receptor (α2‐AR) seems to be a reduced response in pain‐related areas such as the prefrontal lobe, thalamus and amygdala.^12^ And the involvement of basal ganglia in pain regulation has been confirmed. The overactivity of neurons in the globus pallidus, substantia nigra and subthalamic nucleus can lead to the inhibition of the lateral thalamic region, resulting in pain through the disruption of the lateral pain pathway to the parietal and insula lobes and the disinhibition of the medial pain pathway to the anterior cingulate gyrus.^13^

To clarify the role of NE in PD pain symptoms, this study established a 6‐OHDA‐induced rat model of PD. Brain NE content was decreased by DSP‐4 (or anti‐DBH‐saporin) and increased by yohimbine and guanfacine injection. The nociception threshold was detected. The activation of glial cells in rat brain was detected by immunohistochemistry and immunofluorescence. The objective is to investigate the relationship of nociception threshold with NE and to discuss the receptor effect on nociception threshold through the application of adrenergic α2 receptor blockers and agonist.

## 2 MATERIALS AND METHODS

### 2.1 Animals

Healthy male clean‐grade Sprague Dawley (SD) rats were purchased from the Animal Experimental Center of the Second Affiliated Hospital of Harbin Medical University. Rats aged 6–8 weeks and weighing 180–220 g were selected, housed in a quiet environment with 60%–80% humidity and 22 ± 2°C temperature with free water intake, artificial circadian rhythm (12 h light/dark cycle). The procedures and treatment of the animals are strictly in accordance with the rules and regulations of the International Pain Society. This study was approved by the Ethics Committee of Harbin Medical University (KY2018‐230). Of the rats that were verified to meet the requirements, five rats were entered into the analysis for each experimental group.

#### 2.1.1 Preparation of PD rat model using 6‐OHDA

Healthy rats without rotation behavior were selected and anesthetized with 2% pentobarbital sodium (4.5 mg/kg) by intraperitoneal injection. The rat scalp hair was shaved, routine iodophor disinfected, fixed the rat on a stereotaxic apparatus (David, Kopf instruments). Cut through the scalp to expose the skull. A unilateral two‐site injection was made into the striatum. Coordinates of striatum in the right hemisphere: Site 1: 0.5 mm posterior to the anterior fontanel, 4.2 mm right of the midline, and 5 mm subdural. Site 2: 0.5 mm anterior to the anterior fontanel, 2.5 mm right of the midline, and 5 mm subdural, drilling along the marked coordinates with a skull drill, inject 6‐OHDA 4 μL (diluted with 0.9% NaCl at a concentration of 5 μg/μL, containing 0.1% ascorbic acid) into the right striatum with a microsyringe. Leave the needle for 5 min, then remove the needle and close the wound. apormorphine hydrochloride (1 mg/kg) was injected subcutaneously into the neck of the rats 2 weeks later. After 10 min, count the number of contralateral rotation of the rat for 30 min. The successful PD rat model with 7 r/min was labeled as “6‐OHDA group”.

#### 2.1.2 Damage of norepinephric neurons with DSP‐4 or anti‐DBH‐saporin

Norepinephric neuron damage was aggravated by DSP‐4 or anti‐DBH‐saporin. The rats were striatally injected with 6‐OHDA following the procedure described above. The Neurotoxin DSP‐4 hydrochloride (DSP‐4, AMEKO) injection was then continued without intervals. DSP‐4 (1 mL 0.9%NaCl+10 mg DSP‐4) was injected 4 μL into the ventricle 1.5 mm beside the fontanelle, 1.1 mm toward the fontanelle and 4.5 mm deep. Two weeks later, the rotation behavior of the rats was detected. The other group of rats: after 6‐OHDA injection, 4 μL of anti‐DBH‐saporin (Merck) was injected into the right lateral ventricle (1.25 μg/μL, 0.9% NaCl dilution), and injected at the rate of 1 μL/min.

#### 2.1.3 Control group

The control group was striatally injected with 0.9% NaCl containing 0.1% ascorbic acid 4 μL following the procedure described in the preparation of PD model using 6‐OHDA.

### 2.2 Nociception threshold determination

#### 2.2.1 Heat nociception sensitivity experiments

The rats to be measured were placed on a glass plate. After 30 min of acclimatization, the measurements were carried out in a quiet state with plantar hot spot apparatus, and the temperature was set at 50°C. The probe of the hot spot stimulator was placed on the sole of the rat, and the retraction time of the rear claw was recorded. Thirty seconds was the longest termination time. A total of three tests were performed, each interval of 5 min, and the average value was recorded as heat sensitivity threshold (HST). The HST of rats were measured in bilateral hind claws before and 2 weeks after modeling and observation continued until week 4.

#### 2.2.2 Mechanical nociceptive threshold determination

The rats to be measured were placed in a plastic cylinder holder and acclimated to the environment for 30 min. The rear claw of the rat was placed under the stimulation probe of the tenderness instrument, and the pressure value was recorded when the rat retracted foot occurred. The foot of each rat was stimulated three times with an interval of 10 min, and the mean value was taken as the pressure withdrawal threshold (PWT). The detection time of PWT was set at the same time as that of HST.

Measurement of PWT after drug application: Multiple doses of yohimbine and guanfacine were administered at the 4th week after modeling intraperitoneally. The drug was given once every half‐life interval for a total of 7 half‐lives to achieve a steady‐state plasma concentration of the drug (yohimbine 2.5 mg/kg per dose, half‐life 0.5 h. guanfacine 0.12 mg/kg per dose, half‐life 21 h).^14^ PWT was detected before and after administration.

### 2.3 Detecting NE contents by ELISA

The whole brain tissue of the decapitated rats was weighed, and PBS was added at 1:9 (1 g:9 mL) to make the homogenate, then centrifuged 10 min at 4°C (3500 r/min), and the supernatant was retained. The diluted standard product and streptavidin‐HRP was added 50 μL each to the standard well. Add 40 μL of test sample, 10 μL of NE antibody, and streptavidin‐HRP 50 μL to the test sample well. Seal the plate, cover with sealing plate film, and incubate at 37°C for 60 min. After washing, add 50 μL of chromogen A and B to each well. The color was developed for 15 min in the dark at room temperature, the reaction was terminated by dropping stop solution 50 μL. Absorbance (OD value) was measured at 450 nm.

### 2.4 Tyrosine hydroxylase (TH) and GFAP protein was detected by western blotting (WB)

The decapitated rat brains were taken, and the tissues were weighed and ground. The tissue lysate (slb‐p0100‐10, Solaibao) containing protease inhibitors (slb‐pc0020, Beyotime) was added at a ratio of 1:10 (1 g:10 μL). After lysis at 4°C for 30 min and centrifugation (12,500 rpm/min, 15 min), the supernatant was collected and the protein concentration was determined by BCA protein assay (slb‐pc0020, Solaibao). Added protein loading buffer (slb‐p1040, Solaibao), heated at 100°C for 10 min, and stored frozen at −80°C.

Tissue protein samples were separated by polyacrylamide gel (SDS‐PAGE) electrophoresis (JKSJ‐PG11, Yamei). Isolated proteins were transferred to polyvinylidene difluoride (PVDF) membranes at low temperatures (Millipore, ML‐IPVH00010), then sealed with 5% skim dry milk at room temperature for 1 h. The blocked PVDF membrane was incubated in diluted rabbit anti‐TH (Abcam: ab37869, 1:5000) or rabbit anti‐GFAP antibody solution (Signalway Antibody:32033, 1:1000) overnight at 4°C. The next day, the film was removed and cleaned with PBST on the shaker at room temperature for three times. PVDF membrane was placed in goat anti‐rabbit diluent (1:10,000), incubated for 70 min at room temperature, then cleaned with PBST for three times, and the target strip image was obtained using the film scanner (clinx, Ying Xiang chemiluminescence system‐6000). The gray of western bands was quantified using image J software.

### 2.5 Immunohistochemistry

The rats were anesthetized. 0.9% NaCl and 4% paraformaldehyde solution were injected into the left ventricle successively, then decapitate the head and take the brain, fixed with 4% paraformaldehyde for at least 24 h. Brain tissue on the 6‐OHDA injection side was paraffin embedded and sectioned, brain tissue sections from the control group were taken from the sham‐operated side. The tissue sections were incubated overnight with diluted primary antibody (rabbit Anti‐GFAP, 1:200). After rinsing with PBS, anti‐rabbit secondary antibody dilutions were added and incubated for 60 min at room temperature. DAB (Beijing Zhongshan Jinqiao Company) staining was performed for 3–5 min away from light, deionized water was used to terminate color development, hematoxylin was redyed for 2–5 min, then dehydrated with ethanol, deethanolized with xylene, and sealed. Images were taken using a Zeiss axiophot microscope (Carl Zeiss). The GFAP immunohistochemical staining was mainly located in the dendritic part of astrocytes, and the activated astrocytes had thickened dendrites. The cells including the brown and yellow particles were judged as GFAP‐positive cells under high power microscope.

### 2.6 Immunofluorescence

The paraffin‐embedded sections were broken by 0.3% Triton X‐100 for 5 min, soaked in methanol solution containing 3%H_2_O_2_ for 10 min, and washed with PBS. Tissue sections were sealed with 5%BSA for 30 min. The rabbit anti‐GFAP antibody was added dropwise and incubated overnight. Rinse with PBS for three times, add anti‐rabbit FITC secondary antibody (1:200) and incubate at room temperature for 1 h. After cleaned with PBS, the section was sealed with anti‐fluorescence quenching agent and photographed under fluorescence microscope. The nucleus was stained blue by DAPI, and the GFAP‐positive cytoplasm was stained red, and the two were overlapped in one cell as GFAP‐positive cells. For immunohistochemistry and immunofluorescence experiments, the rate of GFAP‐positive cells was calculated as follows: The rate of GFAP‐positive cells = the number of GFAP‐positive cells/the total number of cells in the same field × 100%.

### 2.7 Statistical analysis

Data analysis was performed with SPSS 21.0 and plotted with graphpad Prism 8.0. Image J software was used for image analysis. The Kolmogorov–Smirnov test of the normal distribution of the data was applied to the unit sample, Measurement data were described as “mean ± SD”, and *t* test was used for data conforming to normal distribution. Independent sample *t* test was used for ELISA and immunohistochemistry. *p* ≤ 0.05 was considered statistically significant.

## 3 RESULTS

### 3.1 The establishment of PD rat model and aggravation of NE damage in rat brain

The PD rat model was established with 6‐OHDA injection. DSP‐4 and anti‐DBH‐saporin were used to aggravate the damage of norepinephrine neurons at the same time. After 2 weeks of modeling, the rats showed weakened resistance to arrest, erector hair, the coat fur that appears dirty and yellow, active movement reduced, slow movement, trembling, tail biting and foot biting. After apomorphine hydrochloride injection, the rats could be observed to rotate to the opposite side, and to be joined end to end. Rats treated with DSP‐4 or anti‐DBH‐saporin performed as well as those treated with 6‐OHDA alone.

**FIGURE 1:**
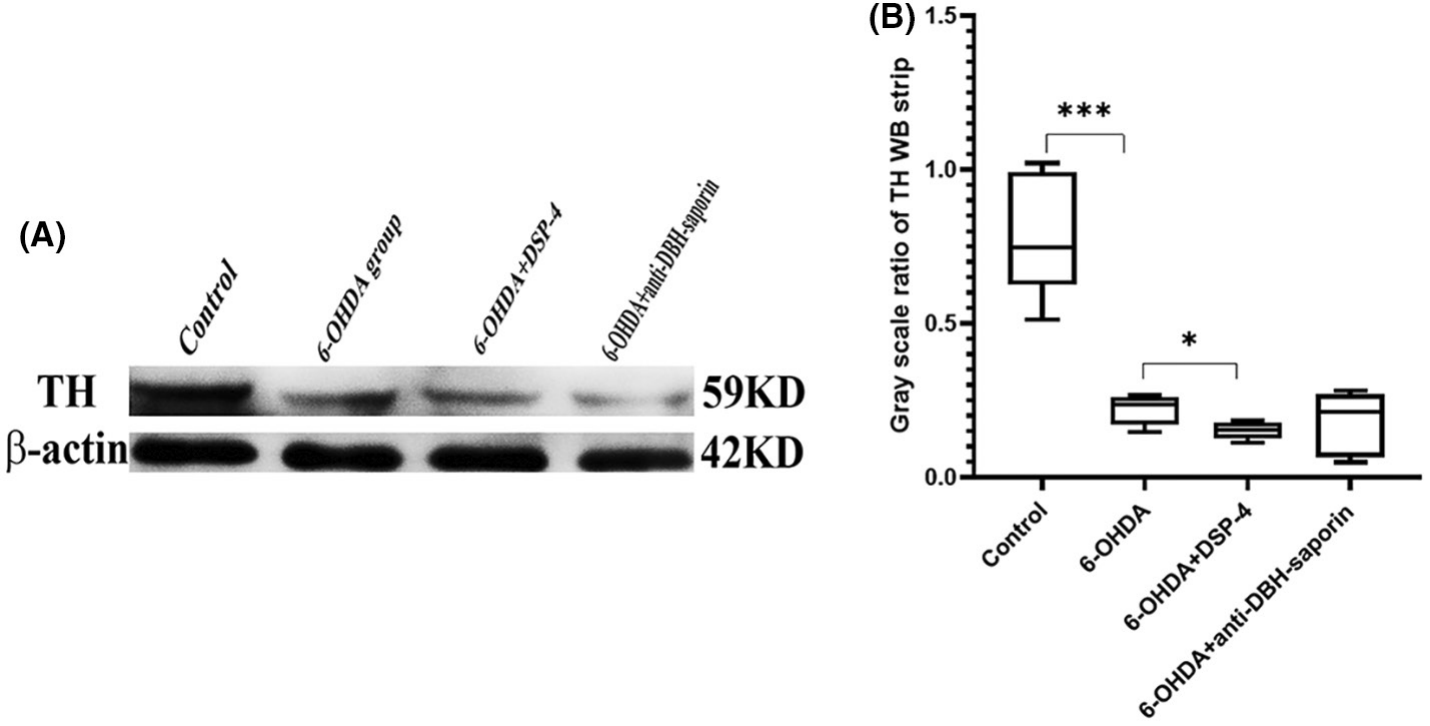
Tyrosine hydroxylase (TH) contents in the striatum of rat models. (A) TH bands of WB in the striatum of the four groups of rat models. (B) The gray ratio of TH/β‐Actin bands in the four groups. TH band of 6‐OHDA group was lower than that of control group, and 6‐OHDA+DSP‐4 group was lower than 6‐OHDA group. There was no static difference between group with DSP‐4 and anti‐DBH‐saporin.*P ≤ 0.05; ***P ≤ 0.001.

The TH content in the bilateral striatum was detected by the methods of western blot (WB). The gray ratio of TH bands of rats in 6‐OHDA group was significantly reduced than that in the control group (*t* = 6.189, *p* = 0.000), and 6‐OHDA group+DSP‐4 group showed lower than that of 6‐OHDA group (*t* = 2.614, *p* = 0.031) (Figure 1A,B). The gray ratio of TH bands was similar between the group with DSP‐4 and anti‐DBH‐saporin (*t* = 0.495, *p* = 0.634).

**FIGURE 2:**
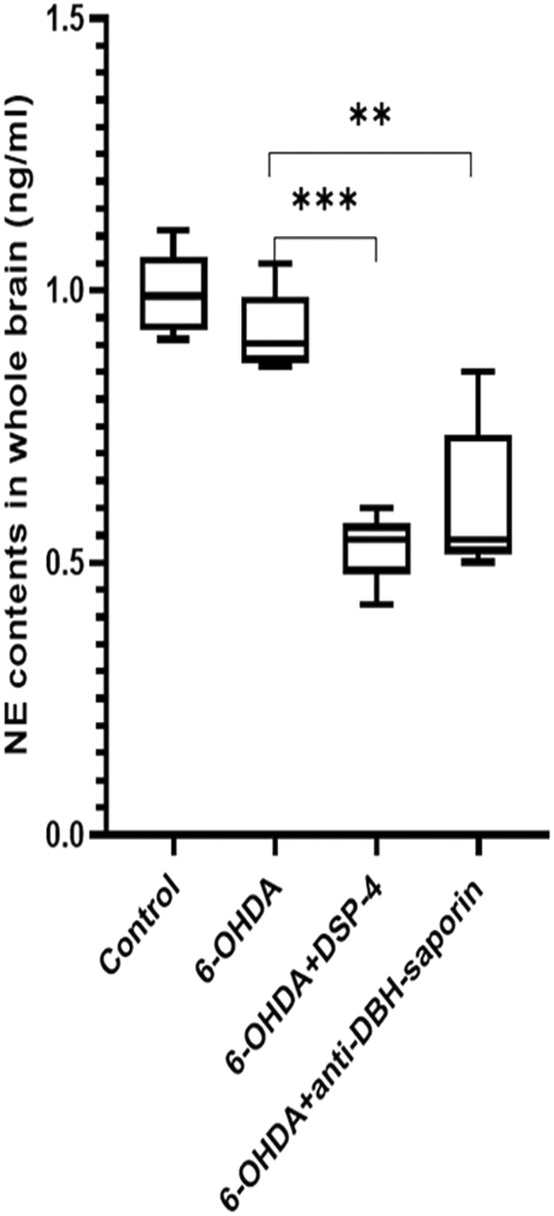
Detection of norepinephrine (NE) contents of the whole rats brain by method of Elisa. The NE content was decreased after using DSP‐4 and anti‐DBH‐saporin compared with the control group and 6‐OHDA group. There was no significant difference between the control group and 6‐OHDA group **p ≤ 0.01; ***p ≤ 0.001.

Norepinephrine contents in whole rat brain were determined by Elisa. NE content of the control group was 0.994 ± 0.077 ng/mL, and there was no statistical difference with 6‐OHDA group (0.923 ± 0.075 ng/mL; *t* = 1.470, *p* = 0.180). The NE content of 6‐OHDA group decreased significantly after DSP‐4 was used (0.528 ± 0.065 ng/mL; *t* = 8.888, *p* = 0.000). There was no significant difference in NE content between DSP‐4 and anti‐DBH‐saporin groups (0.609 ± 0.142 ng/mL; *t* = −1.115, *p* = 0.297; Figure 2).

### 3.2 Evaluation of changes in response to thermal stimulation and mechanical pressure in model rats

**FIGURE 3:**
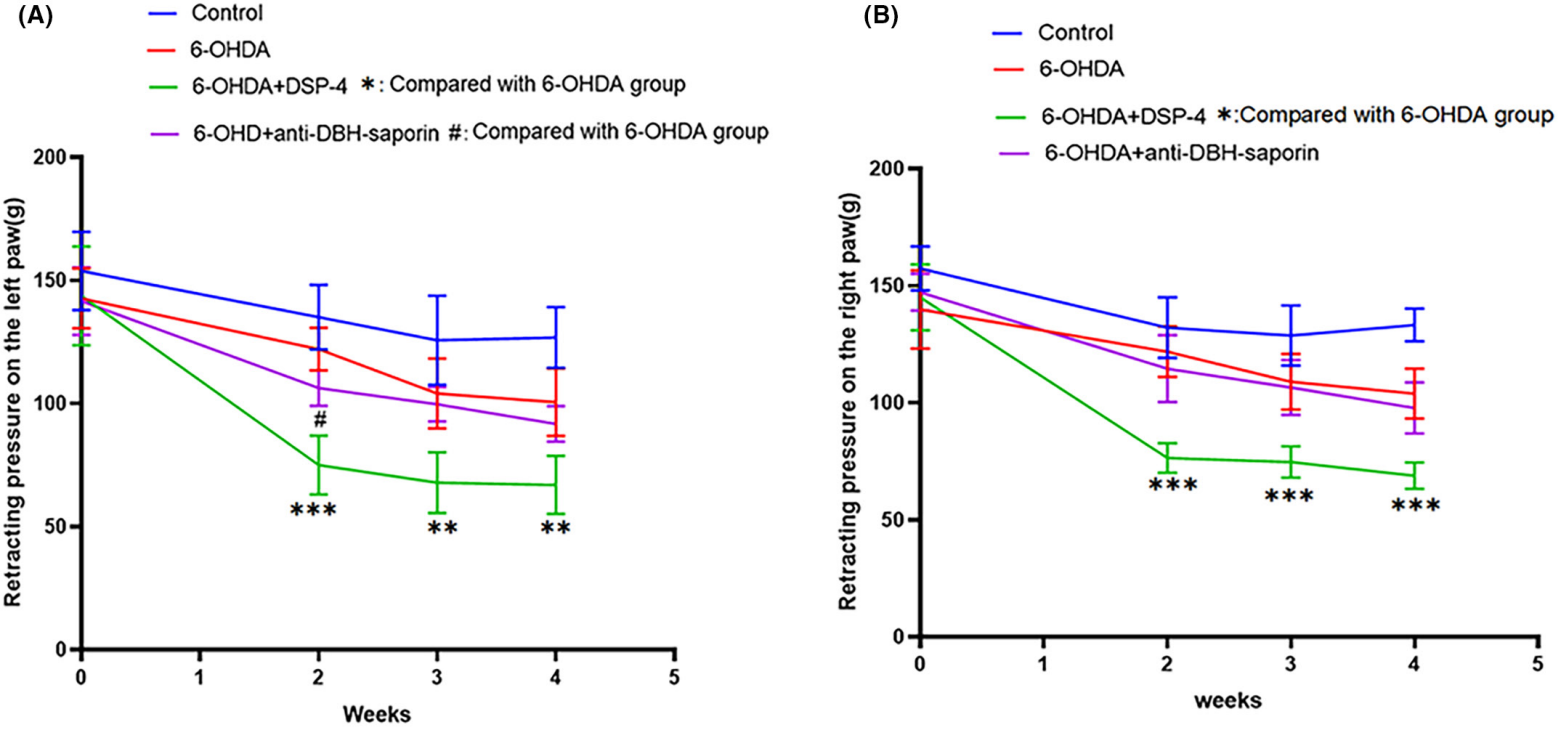
Changes in pressure withdrawal threshold (PWT). (A) The PWT of the left paw. (B) The PWT of the right paw. The PWT of the both posterior paws showed similar results as follows: The PWT of the rat posterior claw in 6‐OHDA group was significantly decreased compared with the control group at week 4. After using DSP‐4, the PWT decreased obviously from week 2 to week 4, and there was a slightly decreased after using anti‐DBH‐saporin.*p ≤ 0.05; ***p ≤ 0.001 (group of 6‐OHDA + DSP‐4 compared with 6‐OHDA group); ^#^ *p* ≤ 0.05 (group of 6‐OHDA + anti‐DBH + saporin compared with 6‐OHDA group).

The results showed that bilateral hind claws responded similarly to mechanical pressure. In the control group, there was no significant change in PWT before and 2 weeks after saline injection, then at the third week (*t* = 2.607, *p* = 0.031) and fourth week (*t* = 2.199, *p* = 0.017), the retraction pressure decreased slightly. The PWT in 6‐OHDA group continued to decrease from week 2 to week 4 and was statistically lower than the control group at week 4 (left: *t* = 3.190, *p* = 0.013; right: *t* = −5.136, *p* = 0.001). After using DSP‐4, the PWT was significantly decreased at each observation time point from week 2 to week 4 (left: week 2: *t* = 7.111, *p* ≤ 0.001; week 3: *t* = 4.284, *p* = 0.003; week 4: *t* = 4.163, *p* = 0.003) (right: week 2: *t* = 8.076, *p* ≤ 0.001; week 3: *t* = 5.604, *p* = 0.001; week 4:*t* = 6.485, *p* = 0.000). However, the stress value after using anti‐DBH‐saporin was slightly lower than that of 6‐OHDA group, and there was a statistical difference between the two groups at the second week (*t* = 3.114, *p* = 0.014; Figure 3).

**FIGURE 4:**
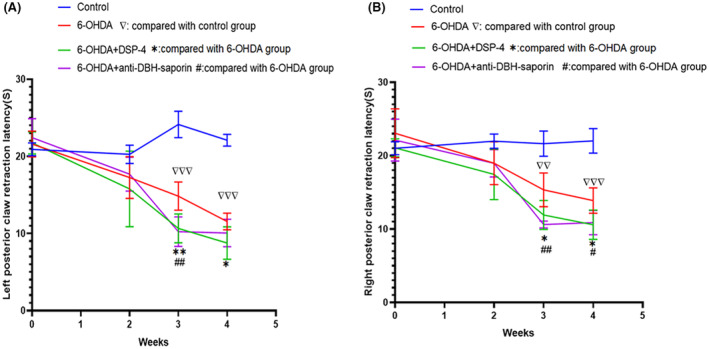
Detection of heat sensitivity threshold (HST). (A) The HST of left claw. (B) The HST of right claw. The result of both posterior PAWS being similar: HST in 6‐OHDA group was significantly decreased compared with the control group, the difference was significant at the third and fourth weeks. HST value after using DSP‐4 and anti‐DBH‐saporin was significantly decreased at week3 and slightly deceased at week 4. *p ≤ 0.05; **p ≤ 0.01 (group of 6‐OHDA + DSP‐4 compared with 6‐OHDA group); ^#^ *p* ≤ 0.05; ^##^ *p* ≤ 0.01 (group of 6‐OHDA + anti‐DBH + saporin compared with 6‐OHDA group).^∇∇^ *p* ≤ 0.01; ^∇∇∇^ *p* ≤ 0.001 (6‐OHDA compared with control).

After 2 weeks of modeling, HST value in 6‐OHDA group continued to decrease until the 4th week, and the downward trend of the left and right hind claw was the same. The HST values of 6‐OHDA group were significantly lower than the control group at week 3 (left: *t* = 8.267, *p* = 0.000; right: *t* = 4.902, *p* = 0.001) and week 4 (left: *t* = 17.775, *p* = 0.000; right: *t* = 7.548, *p* = 0.000). HST values were further reduced after DSP‐4 and anti‐DBH‐saporin at week 3 and week 4 compared with 6‐OHDA group [week3: DSP‐4 group (left: *t* = 3.561, *p* = 0.007; right: *t* = 2.531, *p* = 0.035); anti‐DBH‐saporin (left: *t* = 3.875, *p* = 0.005; right: *t* = 4.513, *p* = 0.002)] [week4: DSP‐4 group (left: *t* = 2.681, *p* = 0.031; right: *t* = 2.785, *p* = 0.024); anti‐DBH‐saporin (left: *t* = 1.590, *p* = 0.150; right: *t* = 2.806, *p* = 0.023; Figure 4)].

Three weeks after saline injection into the right striatum of control rats, the retraction time of the left hind foot in response to temperature stimuli was prolonged. The highest value was found in the third week (24.13 + 1.72 s), followed by the fourth week (22.08 + 0.76 s). Both of them were higher than those in the second week (20.27 + 1.183 s) and the first week (20.90 + 0.84 s), and the difference was statistically significant (HST of the third week vs the first week, *t* = −3.773, *p* = 0.005; HST of the fourth week vs the first week, *t* = −2.343, *p* = 0.047). while the HST changes in the right hind foot were not significant.

### 3.3 Detection of GFAP in the prefrontal lobe, cingulate gyrus and striatum of rats

#### 3.3.1 GFAP detection after the DSP‐4 using

**FIGURE 5:**
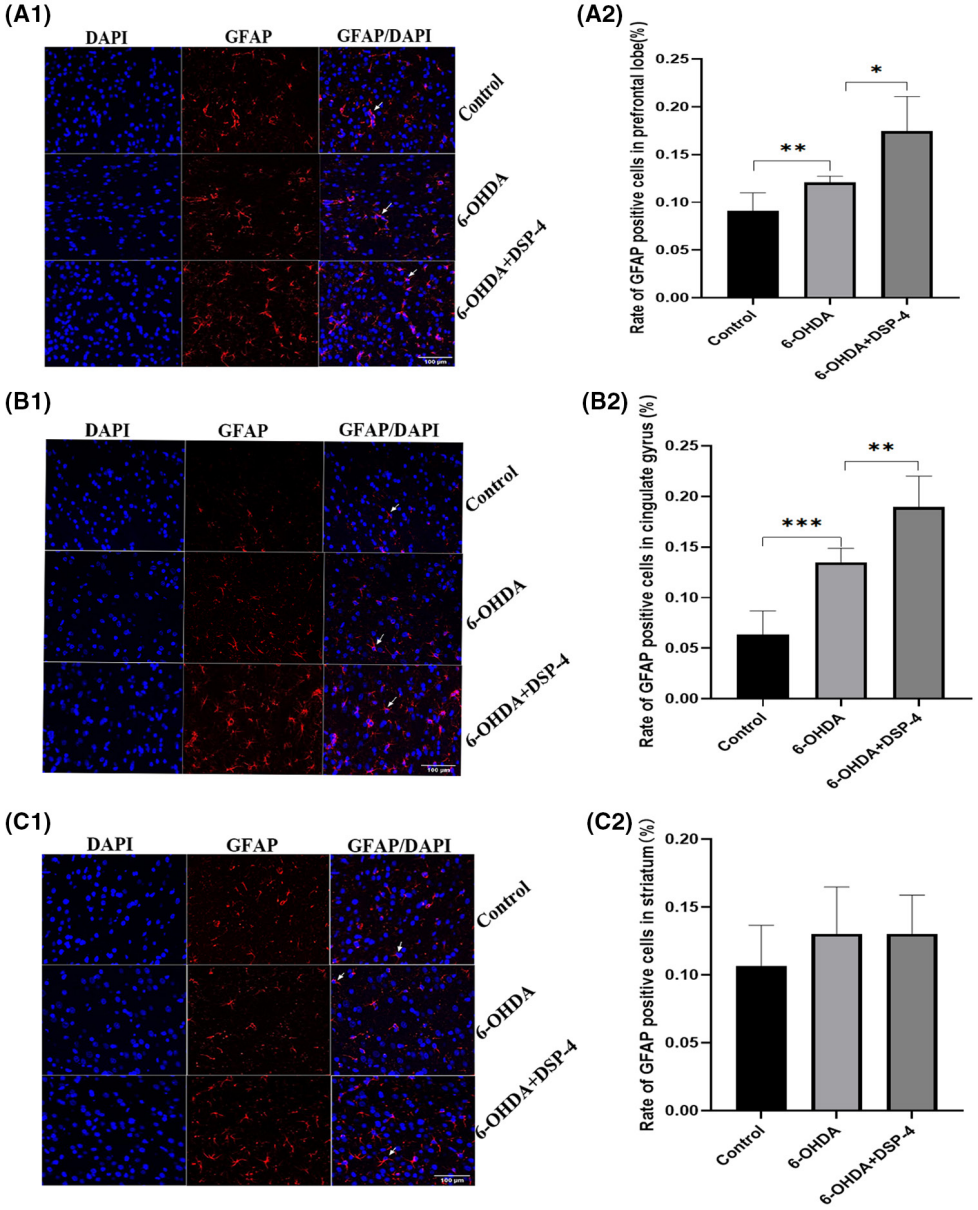
GFAP immunofluorescence staining (400X). (A) GFAP of prefrontal lobe. The ratio of GFAP‐positive cells in 6‐OHDA group was higher than in the control group (*t* = −3.331, *p* = 0.010), and more GFAP positive cells after using DSP‐4 (*t* = 3.250, *p* = 0.012). (B) GFAP staining on anterior cingulate gyrus. The ratio of GFAP‐positive cells in 6‐OHDA group was higher than in the control group (*t* = −5.826, *p* = 0.000), and lower than the group after using DSP‐4 (*t* = 3.634, *p* = 0.007). (C) GFAP on striatum. There was no significant difference among the three groups in the striatum. Right side showed the quantified column of the ratio of GFAP‐positive cells. Blue: DAPI nuclear staining. Red: GFAP staining within the cytoplasm. White arrows refer to the Merge DAPI/GFAP‐positive cells. Scale: 100 μm. *p ≤ 0.05; **p ≤ 0.01;***p ≤ 0.001.

The prefrontal cortex, anterior cingulate gyrus and striatum of rats were selected for GFAP staining by immunofluorescence. The 6‐OHDA group showed significantly higher rates of GFAP‐positive cells in the prefrontal lobe (Figure 5A) and cingulate gyrus (Figure 5B) compared with the control group, after DSP‐4 injection, the rate of GFAP‐positive cells increased. AS to GFAP staining on striatum, the rate of GFAP‐positive cells was higher in 6‐OHDA group and DSP‐4 group than that of control, but there was no significant difference (Figure 5C).

**FIGURE 6:**
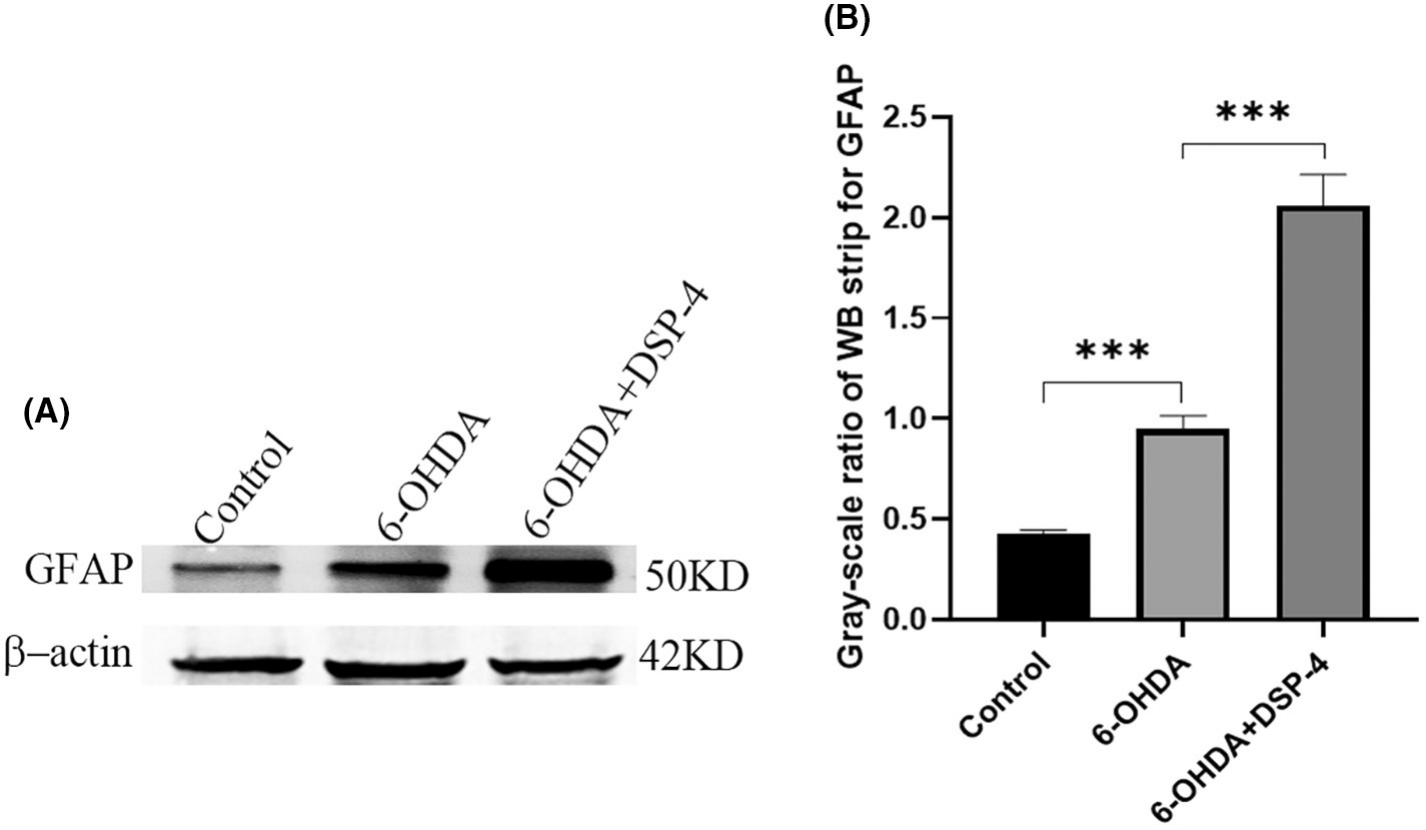
The detection of GFAP protein in the prefrontal lobe by western blotting.(A) GFAP band of the three group. (B) The gray ratio of GFAP band of the three group. The gray ratio of GFAP band (GFAP/β‐Actin) increased in 6‐OHDA group, and further increased after the application of DSP‐4. ***p ≤ 0.001.

To verify the reliability of increased GAFP expression in immunofluorescence, GFAP protein in the prefrontal lobe was detected by western blot (Figure 6). The gray ratio of prefrontal GFAP band (GFAP/β‐actin) was higher in 6‐OHDA group than in control group, and increased after DSP‐4 use.

#### 3.3.2 GFAP detection after using anti‐DBH‐saporin

**FIGURE 7:**
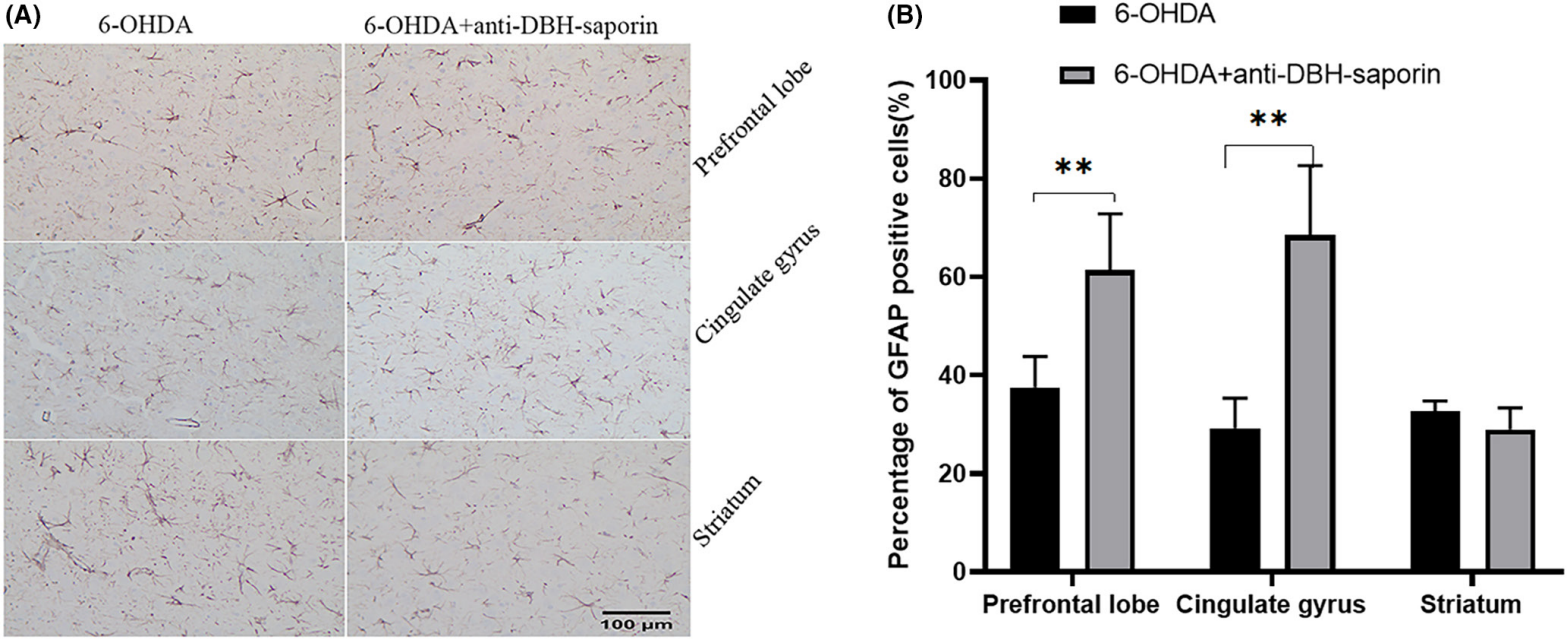
(A) Immunohistochemical staining of GFAP cells (SABC × 200). GFAP is mainly located in dendrites. (B) The ratio of GFAP positive cells. There were more GFAP‐positive cells in the prefrontal lobe and cingulate gyrus after using anti‐DBH‐saporin (*n* = 5). Scale: 100 μm. **p ≤ 0.01.

GFAP after using anti‐DBH‐saporin was detected by the method of immunohistochemical. Compared with 6‐OHDA group, the percentage of GFAP‐positive cells in the prefrontal lobe and cingulate gyrus after using anti‐DBH‐saporin was significantly increased (*p* ≤ 0.01; Figure 7). In the striatum, there was no statistical difference in GFAP‐positive cells between the two groups (*p* = 0.259).

### 3.4 Effect of norepinephrine α2 receptor on PD rats

#### 3.4.1 Change of nociception threshold after yohimbine and guanfacine injection

**FIGURE 8:**
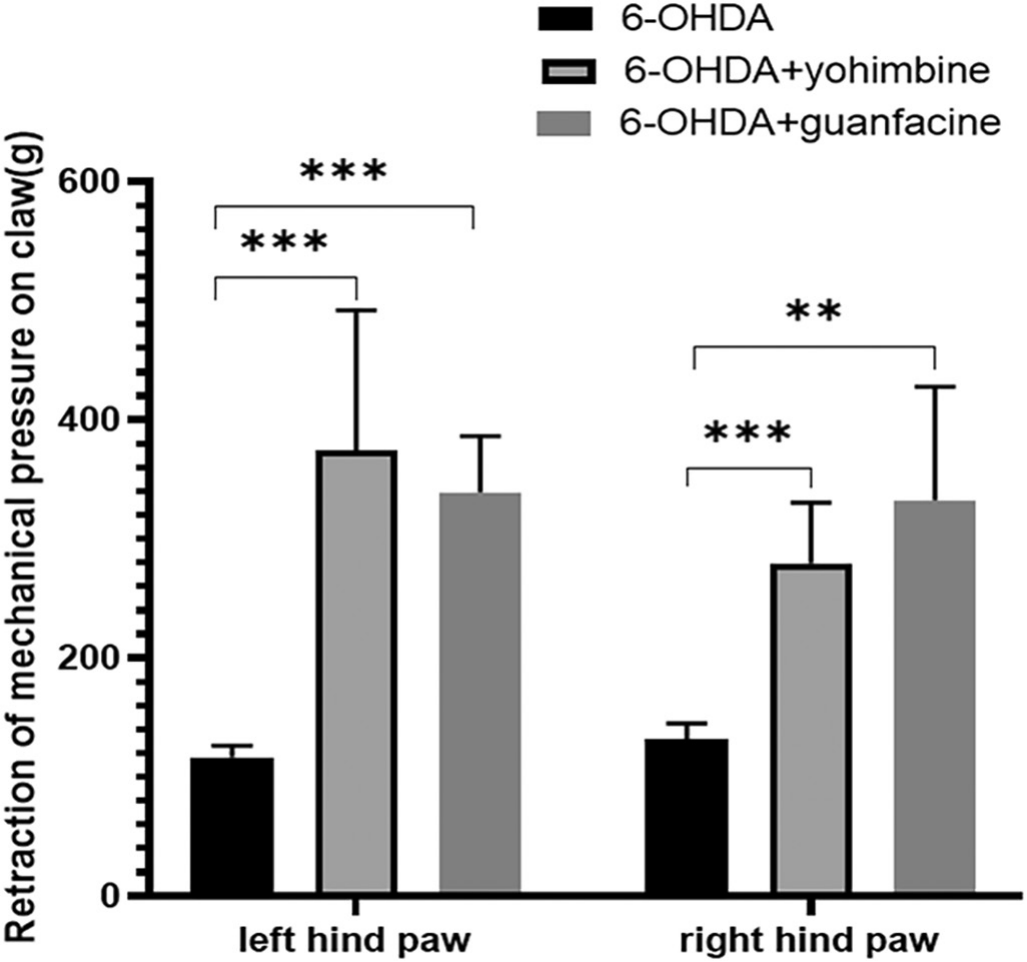
Measurement of pressure withdrawal threshold (PWT) after drug application: The PWT significantly increased after yohimbine and guanfacine injection both in left and right paws. ***p ≤ 0.001, **p ≤ 0.01. *n* = 5/group.

After intraperitoneal injection of yohimbine, the PWT of both posterior PAWS of 6‐OHDA group was increased (left: *t* = −4.895, *p* = 0.001; right: *t* = −6.263, *p* = 0.000). The effect was the same in the group treated with guanfacine intraperitoneally (left: *t* = −10.256, *p* = 0.000; right: *t* = −4.665, *p* = 0.002; Figure 8).

Five weeks after 6‐OHDA injection, the NE contents decreased in the prefrontal lobe and cingulate gyrus compared with control group (Table 1). intraperitonal injection of yohimbine and guanfacine significantly increased NE content in the prefrontal cortex. NE content in cingulate gyrus also increased, but did not reach statistical significance. The NE content of striatum in 6‐OHDA group had no significant difference with that in Yohimbine and Guanfacine group.

**TABLE 1:** Norepinephrine (NE) contents in Parkinson's disease (PD) rat models (*n* = 3, mean ± SD, ng/mL).

Group	Prefrontal lobe	Cingulate gyrus	Stratium
Control	7.093 ± 0.294	4.459 ± 0.563	1.444 ± 0.317
6‐OHDA group	2.581 ± 0.544^a^	3.043 ± 0.634^d^	1.844 ± 0.722
6‐OHDA + yohimbine	6.643 ± 0.877^b^	5.133 ± 1.192	2.345 ± 0.588
6‐OHDA + guanfacine	6.081 ± 0.457^c^	4.749 ± 0.904	2.307 ± 0.594

^a^ Compared with control group, *t* = 12.627, *p* = 0.000.

^b^ Compared with 6‐OHDA group, *t* = 6.815, *p* = 0.002.

^c^ Compared with 6‐OHDA group, *t* = 8.527, *p* = 0.001.

^d^ Compared with control group, *t* = 2.893, *p* = 0.044.

#### 3.4.2 The number of GFAP‐positive cells after yohimbine and guanfacine injection

After intraperitoneal injection of yohimbine or guanfacine, the percentage of GFAP‐positive cells in the prefrontal lobe and cingulate gyrus of rats was significantly decreased compared with that in 6‐OHDA group (Table 2). There was no significant change in the proportion of GFAP‐positive cells in striatum among the three groups.

**TABLE 2:** The rate of GFAP‐positive cells in Parkinson's disease (PD) rat models (*n* = 3, ±SD, %).

Group	Prefrontal lobe	Cingulate gyrus	Stratium
6‐OHDA group	37.554 ± 6.268	29.231 ± 6.092	33.950 ± 0.021
6‐OHDA + yohimbine	12.074 ± 1.833^b^	14.802 ± 1.367^a^	31.510 ± 0.018
6‐OHDA + guanfacine	7.094 ± 1.662^b^	14.689 ± 2.650^a^	40.395 ± 0.287

^a^ *p* ≤ 0.05.

^b^ *p* ≤ 0.01, compared with 6‐OHDA group.

## 4 DISCUSSION

Norepinephrine (NE), first discovered in the brain 60 years ago, is a neurotransmitter synthesized from tyrosine in successive enzymatic reactions. NE can regulate pain through direct or indirect action on different adrenergic receptors, among which, a2 adrenergic receptors widely distributed in the central nervous system may play a major role in the regulation of neuropathic pain. Braak found that the deposition of a‐synuclean began in the locus coeruleus as early as in the preclinical or prodromal stage of PD, the amount of NE in the brain of PD patients is reduced by 80%–90%.^6^ Therefore, we speculated that NE might be involved in the regulation of PD pain symptoms through adrenergic a2 receptor.

In this study, 6‐OHDA was used to establish a classic PD rat model, which mainly reduced dopaminergic neurons in the brain of rats. Then, noradrenergic neuron damage was aggravated by DSP‐4 or anti‐DBH‐saporin injection. After 2 weeks of injection, the three groups of rats showed the same appearance and rotation behavior induced by apomorphine. External features could not determine whether rats had different degrees of norepinephrine neuronal damage, so the content of norepinephrine was detected in the whole brain of the rats. After the application of DSP‐4 and anti‐DBH‐saporin, some rats had decreased NE content in brain, and DSP‐4 appeared to cause more severe damage to NE system than anti‐DBH‐saporin. It was also shown that the TH content decreased after DSP‐4 injection compared with 6‐OHDA group. Previous studies suggested that DSP‐4 can selectively damage noradrenergic neurons, while non‐NE neurons are not damaged.^15^ However, other studies suggest that DSP‐4 may damage 5‐HT neurons as well as NE neurons. In this study, the decrease of TH content not only affects the synthesis of norepinephrine, but also may affect the synthesis of dopamine. However, anti‐DBH‐saporin can selectively destroy NE neurons and hardly affect other monoamines in the brain.^16^ In this study, DSP‐4 was injected intraventricular rather than intraperitoneal. Intraperitoneal injection of DSP‐4 (50 mg/Kg) was also attempted at the early stage of the study, but due to the weight of the rats, the dosage was large, and the survival rate of the rats decreased, while intraventricular injection, the dosage was small, and the survival rate of the rats increased. It also achieved the purpose of reducing NE. Rats with reduced NE content after DSP‐4 or anti‐DBH‐saporin were included in the following analysis.

It was reported that PD rat models induced by 6‐OHDA showed heat and mechanical hypersensitivity, however, additional consumption of norepinephrine by injecting DSP‐4 can further aggravate hypersensitivity to nociceptive stimuli in PD rats.^17^ In this study, the HST and PWT of rats in PD rats were significantly decreased compared with the control group. Although the damage to dopaminergic neurons was unilateral, the decrease in nociception threshold showed the same trend on both sides. It is suggested that the effect of dopamine on nociception threshold is not accomplished by contralateral spinothalamic tract. When DSP‐4 or anti‐DBH‐saporin was applied to increase the damage to norepinephrergic neurons, the nociception threshold of rats decreased more significantly in bilateral hind paws, which were similar to previous reports. Moreover, the rats treated with DSP‐4 had the greatest decrease in nociception threshold. It has been reported that DSP‐4 alone can reduce the NE content in the frontal cortex, hippocampus and hypothalamus to 16.5%, 22.3% and 69.6%, respectively,^18^ and the degree of decrease in NE content is significantly correlated with the shortening of thermal stimulation latency.

The puzzle is that the retraction time of the left hind foot in response to temperature stimuli was prolonged in the control rats. It has been reported that Long‐term muscle exercise, acupuncture, and low‐frequency electrical stimulation can cause the afferent nerve to discharge and affect the central endorphin mechanism to produce analgesia^19^ Another study has also reported that training can reduce nociceptive pain in rats.^20^ So the possibility was considered that repeated measurements of the mechanical and thermal irritation may play a training role in the rats to increase their threshold of nociceptive stimulation.

Noradrenergic nerve fibers from the locus coeruleus to the prefrontal cortex and cingulate gyrus are involved in regulation, cognition and emotional response to pain.^21^, ^22^, ^23^ Damage to norepinephrergic neurons by DSP‐4 and anti‐DBH‐saporin resulted in reduced fibrillary projection from the locus coeruleus to the prefrontal and cingulate gyrus, possibly resulting in altered activity in these brain regions. In this study, immunofluorescence, immunohistochemistry and Western blot were used to detect GFAP in the prefrontal cortex, cingulate gyrus and striatum of rats. The results consistently showed that the ratio of GFAP‐positive cells in the prefrontal cortex and cingulate gyrus increased in 6‐OHDA‐induced PD rats. Moreover, the proportion of GFAP‐positive cells increased further after DSP‐4 and anti‐DBH‐saporin application, which was consistent with the changes of HST and PWT in rats. This indicates that the decrease of NE may promote the activation of glial cells in these two regions, resulting in central sensitization and participating in the regulation of nociception threshold in PD rats. Previous studies showed that astrocytes can activate some key signaling pathways (such as MAPK signaling pathway), synthesize and release inflammatory mediators, sensitize pain sensory neurons, lead to chronic pain.^24^ And there have been non‐Parkinson's studies showing that astrocyte activation in cingulate gyrus is associated with pain.^25^

Most of the a2 adrenergic receptors in the central nervous system are located on the presynaptic membrane of NE neurons. The presynaptic receptor is negatively coupled with adenylate cyclase to activate K+ current and inhibit the presynaptic calcium channel, reducing the release of neurotransmitter NE.^26^ In this study, yohimbine, the presynaptic α2 adrenergic receptor blocker, was injected intraperitoneally into PD rats. The PD rats showed elevated HST and PWT thresholds, increased levels of norepinephrine in their prefrontal lobes and decreased GFAP‐positive cells in the prefrontal cortex after injection. It was confirmed that a2 adrenergic receptors affect nociceptive threshold of PD rats by increasing NE content and altering cell activation. After yohimbine was applied, NE did not increase in the cingulate gyrus statistically, but the percentage of GFAP‐positive cells still decreased, which may be the reason for the small sample size. There was no statistical difference between NE and GFAP‐positive cells in striatum, which may be related to receptor distribution, and it is speculated that receptor‐mediated changes of nociception threshold may not be related to striatum.

Whether increased NE directly bind to the receptors on the surface of glial cell and inhibit glial cell activation? Which receptor is involved? These questions still need to be explored. Otherwise, NE may act on the interneuron, the fibers of the interneuron act on the glial cells. It may be the target of the next discussion. A study has shown that NE can act on the α1 receptor in glial cells, affecting the intracellular calcium ion signal and thus playing a role.^27^

Paradoxically, this study showed that the application of guanfacine, an adrenergic α2 receptor agonist, improved the pain sensitivity in the hind paws of PD rats. This is the same effect as applying yohimbine. Since adrenergic α2 receptors in the rat brain are mainly located in the presynaptic membrane, while guanfacine mainly acts on the postsynaptic membrane, the mechanism of guanfacine action on nociception threshold may be more complicated. In one study, preinjection of clonidine into the hind paw of rats reduced the sensitivity of the sole to thermal stimulation,^28^ it may be that norepinephrine inhibits capsaicin‐induced transient receptor potential vanillin 1 (TRPV1) activity via α2 receptor in rat dorsal root ganglion (DRG) neurons. Mechanical hyperalgesia induced by Fredlin adjuvant (CFA) can be relieved by injection of α2 adrenergic receptor agonists into the psoas major muscle.^29^ Therefore, it is considered that the improvement of algesthesia induced by guanfacine in this study may be achieved through the effect of peripheral adrenergic α2 receptor. The use of guanfacine was also associated with a decrease in GFAP‐positive cells in the prefrontal lobe and cingulate gyrus, just as activation of microglial cells in the spinal cord after peripheral nerve injury may make pain persist. It is speculated that signals from the periphery and spinal cord may affect the activation of brain cells.

Conclusion: Norepinephrine has been linked to nociceptive threshold of PD rats; The change of norepinephrine content can affect the activation of glial cells in prefrontal and cingulate gyrus and participate in the regulation of nociception threshold in PD rats. adrenergic α2 receptor agonist and central presynaptic membrane α2 receptor blocker both affect cell activation and improve hyperalgesia.

## AUTHOR CONTRIBUTIONS

Qing Gao and Yingying Zhang made the equal contributions to this work. Xiaoying Wang, Rui Wang and Limei Zhang made part of the works on the experiment. Limei Zhang was responsible for the design of the subject, data processing and statistical analyses, manuscript drafting and revision. Qing Gao, Yingying Zhang, Xiaoying Wang and Rui Wang was responsible for the experimental part of the subject, data analysis, drafting of the article.

## CONFLICT OF INTEREST STATEMENT

The authors declare no conflicts of interest.

## Data Availability

The data can be made available upon reasonable request by a qualified researcher.
